# Electrodermal Activity Analysis at Different Body Locations

**DOI:** 10.3390/s25061762

**Published:** 2025-03-12

**Authors:** Patricia Gamboa, Rui Varandas, Katrin Mrotzeck, Hugo Plácido da Silva, Cláudia Quaresma

**Affiliations:** 1 LIBPhys (Laboratory for Instrumentation, Biomedical Engineering and Radiation Physics), NOVA School of Science and Technology, 2829-516 Caparica, Portugal; r.varandas@campus.fct.unl.pt; 2 PLUX—Wireless Biosignals S.A., 1050-059 Lisbon, Portugal; katrin.mrotzeck@gmail.com (K.M.);; 3 IT—Instituto de Telecomunicações, 1049-001 Lisbon, Portugal

**Keywords:** electrodermal activity, alternative site, skin conductance response, skin conductance level

## Abstract

Electrodermal activity (EDA) reflects the variation in the electrical conductance of the skin in response to sweat secretion, constituting a non-invasive measure of the sympathetic nervous system. This system intervenes in reactions to stress and is strongly activated in emotional states. In most cases, EDA signals are collected from the hand (fingers or palms), which is not an ideal location for a sensor when the participant has to use their hands during tasks or activities. This study aims to explore alternative locations for retrieving EDA signals (e.g., the chest, back, and forehead). EDA signals from 25 healthy participants were collected using a protocol involving different physical stimuli that have been reported to induce an electrodermal response. The features extracted included the Skin Conductance Response (SCR) height, SCR amplitude, and peak prominence. An analysis of these features and the analysis of the correlation between the standard position with the different locations suggested that the chest, while a possible alternative for EDA signal collection, presents some weak results, and further evaluation of this site is needed. Additionally, the forehead should be excluded as an alternative site, at least in short-term measurements.

## 1. Introduction

The Autonomic Nervous System (ANS) controls most of the body’s visceral functions [1], innervating the endocrine glands, the exocrine glands (e.g., sweat glands), and the viscera. It is divided into the sympathetic and parasympathetic nervous systems and operates involuntarily through autonomic reflexes and central control [2]. Electrodermal activity (EDA) is a manifestation of the activity of the eccrine sweat glands, which are innervated by the ANS [3]. The EDA is considered to be a peripheral indicator of sympathetic activation [4], and it refers to the variation in the electrical conductance of the skin in response to sweat secretion [5].

The EDA response is categorized by a tonic component and a phasic component. The tonic component refers to gradual and soft changes in the EDA response, which occur in the absence of stimuli [6]. The most common measure of this component is the Skin Conductance Level (SCL). Research suggests its variations seem to reflect the global changes in the autonomic excitation and occur typically in a period of dozens of seconds to minutes [6], in the absence of stimuli [7]. On the other hand, the phasic component, designated by the Skin Conductance Response (SCR), refers to sudden and rapid changes in the EDA response [6]. These phasic responses of conductivity seem to correspond to arousal states, with SCR amplitudes providing information on the intensity of those states [8]. The EDA signal reflects a combination of different processes—attentional, affective, motivational [1]—and it has been used in different studies targeting emotional arousal [9] and stress [10].

The human body contains between 1.6 and 4.0 million eccrine sweat glands in total, with densities per square centimeter of 64 on the back, 181 on the forehead, 600–700 on the palms and soles (Sato et al., 1989, as cited in [11]), and 20 on the chest (Wilke et al., 2004, as cited in [12]). Most researchers use the palms or the volar surfaces of the fingers as active sites for EDA recording, and this is the preferred Standard Position (SP) for EDA collection [11]. However, as the hands are usually used in tasks or activities performed during experimental studies or daily monitoring, research has been conducted to investigate EDA signals retrieved from different body locations.

Several studies have investigated the relationship between the signal retrieved from the fingers (SP) and other body locations, such as the wrist [13,14], feet [15,16], or ankle [13]. However, few studies explore several locations simultaneously.

One study investigated SCR in the context of emotion elicitation through the visualization of emotional film clips, comparing 16 different recording positions in 17 participants [17]. The highest SCL and SCR were observed for the forehead, foot, fingers, and shoulders. Conversely, the lowest SCR was found for the arm, armpit, thigh, buttock, back, and abdomen. The highest correlations between the fingers (SP) and other positions were found for the foot, followed by the forehead, which was among the top three most responsive body locations for SCL and SCRs. Thus, the authors suggest that sensors could be embedded into headbands or headphones to unobtrusively measure EDA. However, in addition to a small sample size, the study also had an imbalanced gender ratio (five females).

Another study compared multiple nonpalmar sites (e.g., the wrist, abductor hallucis of the foot, foot arch, toes, and forehead) with the fingers [18], during the visualization of 19 images from the International Affective Picture System (IAPS; Lang, Bradley & Cuthbert, 2008, as cited in [18]) and an arithmetic stress task. The results revealed that nonpalmar sites are generally less responsive, with the wrist providing the lowest SCL values and the toes the highest, obtaining SCL values closer to the ones obtained from the fingers. Within-participant correlations between the fingers and other sites were higher for the plantar sites and lowest for the forehead, followed by the wrist [18]. In conclusion, toes are the most equivalent alternative in terms of responsiveness to stimuli, followed by the abductor hallucis location (recommended by Boucsein, 2012, as cited by [18]).

In another study, 115 participants performed a breathing exercise (4 min long) and listened to four musical segments (conveying different emotions) and one emotionally neutral computer-generated tone (lasting 7 s each), while EDA was measured from five anatomical sites bilaterally (finger, foot, wrist, shoulder, and calf [19]). The response magnitudes of SCRs to breathing exercises, music segments, and neutral tones were higher at the feet (most likely due to the high density of eccrine sweat glands). Within-subject correlations were also higher for the feet, followed by the wrists (when comparing these locations with the fingers), in all tasks. In summary, among the sites explored in this study, feet are the recommended alternative location for EDA collection. Even though lower SCR amplitudes were found for the wrists (when compared to the fingers), authors recommend this alternative site if the feet are not available. Furthermore, they also highlight that with adequate hydration time (20 min), the calves become comparable to the wrists in terms of response frequency, magnitude and correlation [19]. However, the sample was composed predominantly of females (*n* = 89). Another limitation identified is related to the hydration time, which might be shorter than the amount of time needed for the alternate sites to become electrodermally active. Also, the wide range of ambient temperature in the experiment may have affected the results [19].

A more recent study compared the SP with three other body locations (forehead, neck and foot) in 23 participants [20]. A high correlation between EDA signals from the SP and the foot was obtained, even when analyzing the phasic and tonic components separately. Again, the authors highlight the foot as the best alternative location for EDA acquisition. Moreover, the forehead was considered to be the most robust against motion artifacts and, with adequate hydration (although this could be an issue for short-time applications), it may become more responsive and provide a more accurate SCR. One limitation of this study is the gender balance of the sample (four females), which makes it difficult to generalize the findings.

The present study focuses on analyzing EDA collected in different body locations (forehead, back, and chest) and comparing these signals to the ones retrieved at the SP for EDA acquisition (fingers on the non-dominant hand). The motivation for this study was set in the context of selecting an alternative EDA site, other than the SP, for the collection of EDA signals from Medical First Responders participating in the H2020 project MED1stMR (Medical First Responder Training using a Mixed-Reality Approach featuring haptic feedback for enhanced realism—is an H2020 project that developed scenarios of mass casualty incidents to train Medical First Responders in several different skills, including first triage. The project involved the collection of participants’ biosignals, including EDA and ECG signals (https://www.med1stmr.eu/, accessed on 16 December 2024)), since the participants were required to wear gloves on their hands, which were used to command avatars in a virtual reality scenario (therefore, it was not feasible to have EDA sensors on the hands or fingers as well).

Nonetheless, results can be extended to support studies where the hands are used when performing tasks or activities (using a specific item, writing, etc.). This research includes body locations that are less commonly studied in EDA research, and thus holds relevance for validating the reliability and consistency of EDA signals from non-standard sites. Determining whether EDA can be reliably measured at alternative sites is essential for facilitating unobtrusive biosignal collection. This could significantly improve device usability and enable the deployment of portable devices to be used with various populations and settings.

The novelty of this study lies in its exploration of less commonly studied body locations for EDA measurement, addressing a gap in the literature regarding non-standard EDA collection sites. Furthermore, by examining whether EDA can be reliably measured at alternative sites, the study contributes to the development of more unobtrusive biosignal collection systems, which is particularly valuable for scenarios where hand usage is restricted (e.g., writing, using tools). This work has practical implications for improving wearable device usability, facilitating the deployment of portable technologies across diverse populations and settings.

## 2. Materials and Methods

### 2.1. Data Collection

The physiological data were collected using biosignalsplux acquisition devices from PLUX (PLUX—Wireless Biosignals, S.A., Lisbon, Portugal, https://www.pluxbiosignals.com/, accessed on 16 December 2024), at 10 Hz and 16-bit resolution, with pre-gelled Ag/AgCl electrodes. The EDA data were collected using an exosomatic approach, with an external constant current applied between two electrodes.

EDA data were collected from four different body locations: the fingers, forehead, back and chest. The sample comprised 25 healthy participants (aged 18–51 years old, M = 29.3, SD = 8.9; 14 females). The data collection was performed in an area specifically designated for this purpose, namely a room containing two researchers and each participant. This study was approved by the Ethics Committee of the NOVA School of Science and Technology (protocol code CE-FCT-006-2022).

The inclusion criteria were as follows: aged above 18 years old; no pathology associated; alcohol consumption limited to no more than two times per week (as alcohol is a known psychotropic depressant of the central nervous system [21]); no consumption of psychotropic drugs; no medication (except occasionally); no caffeine consumption in the three previous hours (as caffeine intake leads to elevated electrodermal activity [22]).

**Figure 1:**
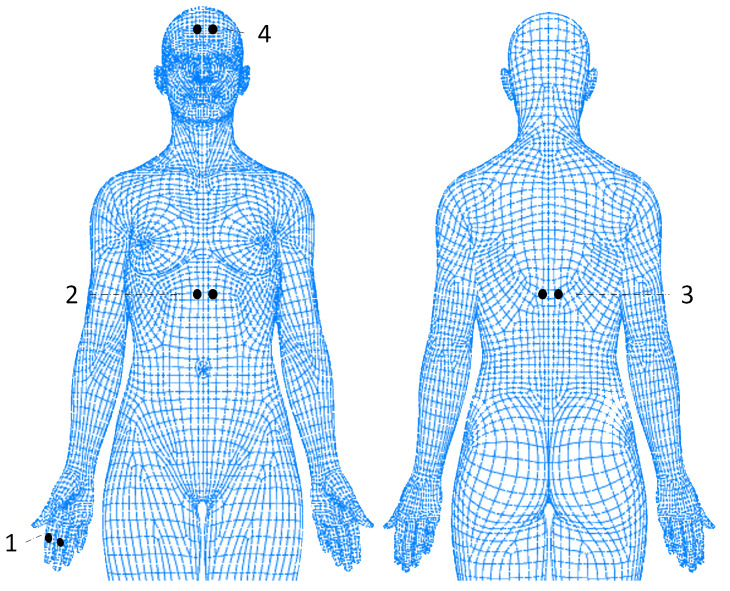
Electrodes positioning (1—fingers of non-dominant hand; 2—chest; 3—back; 4—forehead).

To all participants that met the inclusion criteria and gave consent for their participation, the following protocol was applied. First, the study was briefly explained and informed consent was collected. Then, participants answered questions related to the sample characterization and to their health and well-being. Afterwards, each pair of EDA electrodes was positioned in the following locations (see Figure 1):

1. On the hand (SP/gold standard): one of the electrodes was placed on the proximal phalange of the index finger and of the middle finger, on the non-dominant hand;
2. On the anterior face of the torso (chest): the two electrodes were placed next to each other, on the *Rectus Abdominis*, at the sternum level;
3. On the posterior face and superior part of the torso (back): the two electrodes were placed next to each other, on the inferior zone of the trapezius muscle;
4. On the forehead: the two electrodes were placed next to each other, on the frontal area, approximately 2 cm above the procerus.

To ensure consistency in electrode placement and facilitate reliable comparisons across participants, we selected a specific location on the forehead—2 cm above the procerus—for all measurements.

After placing the electrodes, a performance test to verify the correct positioning and respective visualization of the EDA signal was performed, followed by a 5 min sample acquisition period to increase the electrodermal contact with the sweat glands ducts [23].

The experiment itself included a 3 min EDA signal acquisition to characterize each individual baseline and the EDA signal acquisition during the performance of tasks that induced an electrodermal response. These tasks were performed by one of the researchers, who very carefully applied the materials/items to the skin surface of the subject to allow them to feel the physical sensation of touching different materials, so that the subject did not need to move. The items/materials used for touch sensation included a mug with hot water inside; a cooling pad; sandpaper; cotton; and a needle (stimuli that generate an electrodermal response according to [24]). An additional task—holding breath—was added [23].

The acquisition of the EDA signal from the four different locations was not performed simultaneously. Each signal was acquired using a different device to avoid any potential mutual interference between the signals, using a customized sync cable to enable the acquisition of signals (using two devices) while ensuring precise temporal synchronization of the recorded data.

The experience consisted of three rounds: SP was compared with the chest; then SP was compared with the back; and lastly, SP was compared with the forehead. There was a baseline period of 3 min before each round and tasks were applied in a randomized sequence for each round. Each of the tasks was interpolated by a 30 s period of rest.

After the data collection, electrodes were removed, and the areas where they were placed were cleaned.

**Figure 2:**
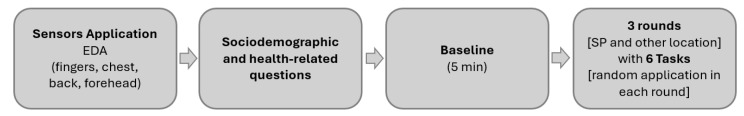
Diagram of the experimental procedure: from sensor attachment to task performance.

The diagram presented in Figure 2 summarizes the protocol described above.

### 2.2. Data Processing

The acquired EDA signal was filtered using a low-pass filter with a bandwidth of 0–3 Hz, as specified in the sensor datasheet (https://support.pluxbiosignals.com/wp-content/uploads/2021/11/Electrodermal_Activity_EDA_Datasheet.pdf, accessed on 16 December 2024). We have also applied a bandpass filter with a bandwidth of 0.045–0.25 Hz.

**Figure 3:**
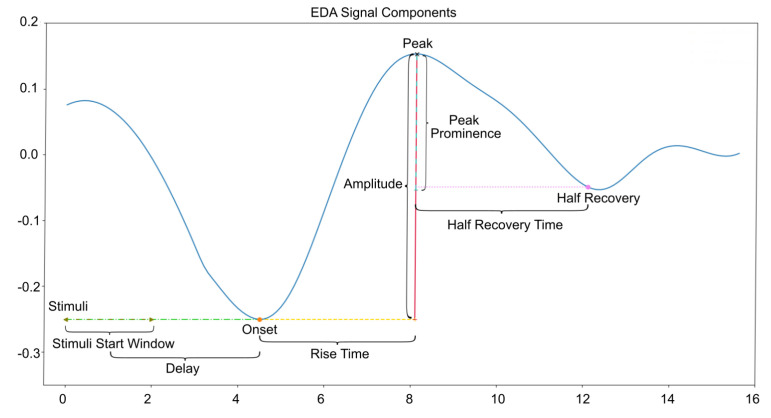
Typical EDA signal response pattern and relevant features.

The tools used for EDA analysis included Python 3.10.9 (Anaconda, Inc., Location Austin, TX, USA) programming language using the NeuroKit2 [25] and SciPy (https://scipy.org/citing-scipy/, accessed on 16 December 2024) packages. Extracted features included the SCR onset, SCR latency or rise time, SCR peak amplitude, and SCR peak prominence (see Figure 3).

SCR height includes the tonic and phasic components, while SCR amplitude excludes the tonic component. SCR peak prominence was also calculated, and it measures how much a peak rises above the surrounding baseline of the signal. It represents the vertical distance between the peak and its lowest contour line. The threshold 0.02 µS was chosen to be the same as in [17], so that the results could be compared more directly, and also to consider the literature that suggests thresholds between 0.015 µS and 0.3 µS [26]. This threshold was considered as the minimum value for each response. Everything below this threshold was ignored to avoid detection of features that were caused by non-task-related artifacts such as movements. Specific time windows in which stimuli were applied were analyzed. For the correlation analysis, we considered the complete time window, whereas for the SCR analysis, we only considered the first peak appearing after applying the stimuli.

## 3. Results

### 3.1. SCR Results by Location

After applying the NeuroKit2 EDA tool [25] to the time windows of the events, we extracted the features for all peaks that appeared within the time windows that were above the mentioned threshold. Afterwards, we only considered the first peak of each time window, averaging the results across all participants with the same sensor position and task. The primary rationale for considering only the first peak was our interest in examining the EDA feedback immediately following the initial stimulus, as this event’s time point could be precisely defined, unlike subsequent stimuli/events within the same time window. Furthermore, this approach was chosen to enhance comparability across different tasks and sensor positions, recognizing that each stimulus might elicit distinct responses over time and a different number of detectable responses. By isolating the first peak, we aimed to ensure consistency and reliability in our analysis across conditions.

Table 1 displays the SCR results by position, presenting the mean value (in µS) and standard deviation in brackets, for the following features: height, amplitude and prominence.

**Figure 4:**
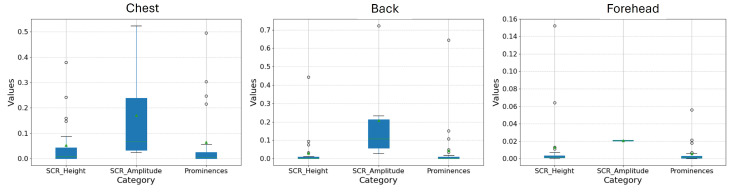
SCR Height, SCR Amplitude, and SCR Prominence per location.

As shown in Table 1, the chest was the location for which the values obtained were closer to the SP in the three features (even though the results were lower than the ones obtained for the SP) . The forehead was the position that resulted in the lowest values of all the positions in all features. Figure 4 depicts a boxplot comparing the positions, regarding SCR Height, SCR Amplitude, and SCR Prominence.

Regarding the amplitude, the peak amplitude of the SCR appears to be the feature that reflects values closer to the SP value (0.17 µS on the chest and 0.21 µS on the back). This suggests that peak amplitude may be a reliable feature for comparing physiological responses across different sensor placements and stimulus conditions.

### 3.2. Correlations with the Standard Position

The correlations between the EDA signals collected at the SP (fingers) and the alternative locations (chest, back, and forehead) were calculated using Pearson’s correlation coefficient [27] for both the tonic and phasic components of the EDA, as presented in Table 2.

As indicated in the table, the EDA signals recorded from the chest exhibit a higher correlation with those collected at the SP, for both the phasic and tonic components, in comparison to other locations, for all tasks except the breathing task.

On the other hand, with the exception of the hot water task, the forehead displayed lower and, in some cases, negative correlations with the EDA from the SP, for both the phasic and tonic components of the EDA.

When computing the mean value for all tasks for each position, the chest yielded the highest values for both the tonic (0.325) and phasic (0.261) components of the EDA. In contrast, the back produced notably lower values (0.176 for tonic and 0.137 for phasic) and the forehead presented the lowest values (−0.005 for tonic and 0.098 for phasic).

These results suggest that, among the alternative measurement sites analyzed, the chest demonstrates relatively stronger signal responsiveness, while the back indicates weaker EDA responsiveness, and the forehead, with a near-zero tonic value and the lowest phasic value, appears to be the least effective site for EDA measurement.

**Figure 5:**
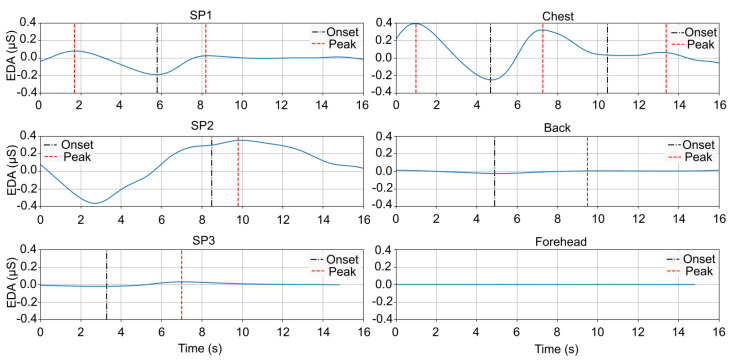
Phasic component of hot water task, for the different locations, for one of the participants.

Figure 5 presents the phasic component around the first peak of the hot water task, for the different locations (SP1—SP round 1—and chest; SP2—SP round 2—and back; SP3—SP round 3—and forehead, respectively), for one of the participants.

The EDA response for the chest follows a similar trend to SP1, showing a clear onset and peak. On the other hand, the back shows a much weaker EDA response compared to SP2, with the signal remaining nearly flat. The forehead EDA signal is flat, with no clear onset or peak. These findings seem to support the exclusion of the back and forehead as viable sites for EDA measurement.

## 4. Discussion

This study explored EDA signal collection from multiple locations on the body, specifically the chest, back, and forehead, comparing them to the standard position (fingers). The objective was to determine viable alternative locations for EDA measurement, particularly when the hands are unavailable due to task performance. Our findings provide meaningful insights into the suitability of alternative EDA collection sites, contributing to advancements in wearable sensor technology and real-world biosignal monitoring applications.

The chest showed relatively better suitability for EDA collection compared to the back and forehead, based on several key indicators. First, it consistently demonstrated signal features that approximated those of the SP across the analyzed parameters, including SCR height, amplitude, and prominence. Secondly, the chest also presented the highest correlation values with the SP for both tonic and phasic components (Table 2), suggesting that it may capture autonomic nervous system responses with acceptable reliability.

Conversely, the forehead consistently produced low and sometimes negative correlations, indicating that this site may not be reliable for EDA collection in short-term applications. Factors such as lower sweat gland density likely contributed to this reduced performance. These findings align with previous research suggesting that forehead EDA signals require specific conditions, such as extended hydration periods, to produce reliable measurements [20]. However, unlike our findings, previous research reported a moderate correlation between EDA measured at the forehead and finger EDA, with the correlation being notably lower for the phasic component [20]. In [17], this site also recorded the highest SCL and the second-highest SCRs value among the 16 locations explored. Regarding correlation with the SP, amongst the 15 alternative sites explored, the forehead ranked fifth highest, while the chest placed ninth, still showing a moderate positive correlation. The back ranked 14th, displaying a low–moderate positive correlation.

In our study, the back also showed poor performance, with correlations lower than those of the chest but still higher than those observed for the forehead. Its performance was task-dependent, particularly in response to thermal stimuli such as the hot water task. This variability suggests that the back may be conditionally useful, depending on the specific monitoring context. Further investigation into task-dependent variability and its influence on sensor reliability at different body locations should be developed.

Considering the results presented in Table 1 and Table 2, we suggest that, while the chest may serve as an alternative site for EDA data collection, its reliability remains limited. Additionally, the forehead should be excluded as a suitable alternative location, particularly for short-term biosignal acquisitions.

A comparison of experimental methodologies reveals differences between our protocol and the one followed by [20], where participants had to relax in a supine position, perform the Stroop Task (a neuropsychological test used to assess the ability to inhibit cognitive interference [28]), walk at 3mph, and lift a dumbbell (each task lasting 120 s). We focused on collecting EDA data while participants experienced the sensation of different materials/items coming into contact with the skin. While these had been reported as stimuli that generate electrodermal response [24], our findings suggest that certain tasks were more effective at inducing noticeable responses than others. Notably, the hot water task consistently generated strong responses across all sensor positions, while the sandpaper and pin tasks were particularly effective when sensors were positioned on the chest. These results highlight the variability in response intensity depending on the nature of the stimuli and sensor placement. Future studies should explore alternative, non-harmful stimuli that may generate more robust and consistent electrodermal responses, contributing to the refining of methodologies for studying EDA.

Ensuring gender diversity in research samples is another fundamental aspect to ensure that we reach conclusions that can be generalized and that are inclusive. In our study, particular attention was given to achieving a balanced sample to ensure adequate female representation. Indeed, 56% of our sample was composed of women, addressing a common limitation found in similar studies (e.g., [20]) and strengthening the relevance and applicability of our results across genders.

Finally, our study was not conducted without some limitations. The sample size was relatively small and derived from a convenience sample, which may limit the generalizability of the findings. Furthermore, EDA acquisitions were conducted sequentially rather than simultaneously, leading to variability in SP values across different rounds of data collection, conditioning the comparison of results between location sites.

Future research should focus on collecting data from larger sample sizes using randomized sampling techniques and standardizing acquisition protocols to improve the reliability and comparability of results. Increasing the sample size would also enable more detailed analyses of demographic variables such as gender and age, thereby expanding the understanding of EDA signals and their variability. This is particularly relevant for enhancing the accuracy and robustness of applications in relation to stress monitoring and emotion recognition. By accounting for demographic factors, these applications could be refined to deliver more personalized and context-sensitive assessments, thereby improving their effectiveness in real-world contexts, including mental health monitoring, workplace productivity, and adaptive human-computer interaction systems.

Several practical and methodological aspects emerged during this study. The decision to analyze only the first SCR peak following each stimulus minimized signal contamination from overlapping responses, ensuring that the results primarily reflected initial autonomic responses rather than cumulative effects. However, future studies should consider multi-peak analysis to capture more complex response patterns. The sequential rather than simultaneous data acquisition may have constrained the generalizability of the findings; thus, future studies implementing simultaneous multi-site recordings would improve data reliability. Additionally, incorporating a wider range of tasks and environmental conditions would provide a more comprehensive evaluation of alternative EDA measurement sites. Future studies could also aim to explore EDA measurement in more naturalistic settings to complement our findings. Finally, future research could incorporate simultaneous respiratory monitoring (using inductive respiration or accelerometer signals) to quantify and account for signal influences due to breathing frequencies.

In summary, the chest shows some potential as an alternative site but still presents a weaker response than the standard finger placement. More research is needed to further characterize the chest as a feasible alternative site for EDA measurement. This alternative placement would be important, especially in scenarios where hand-based monitoring is impractical. The back may serve as a complementary site, particularly when task-specific responses are considered. The forehead, however, appears unsuitable for short-term EDA monitoring due to its inconsistent signal quality. These findings can contribute to the development of more versatile wearable biosensors and expand the possibilities for real-world EDA monitoring in contexts such as stress detection, human–computer interaction, and rehabilitation therapies.

## 5. Conclusions

This study collected EDA signals from three different body locations (chest, back, and forehead) and compared them to the finger EDA, which is considered the standard position for EDA collection. To the best of our knowledge, this is the first study to explore EDA from different sites using physical stimuli that induce electrodermal activity, with a gender-balanced sample. Based on the results, we conclude that although the chest may serve as an alternative site for EDA collection, it is not an ideal replacement for the standard finger placement. Additionally, the forehead should be ruled out as a viable site, particularly for short-term measurements.

## Figures and Tables

**Table 1:** SCR results by location (in µS).

Location	SCR Height	SCR Amplitude	SCR Prominence
SP	0.19 (0.31)	0.34 (0.48)	0.26 (0.43)
Chest	0.05 (0.10)	0.17 (0.18)	0.07 (0.13)
SP	0.33 (0.44)	0.75 (0.99)	0.40 (0.52)
Back	0.03 (0.09)	0.21 (0.24)	0.04 (0.13)
SP	0.06 (0.06)	0.10 (0.09)	0.07 (0.08)
Forehead	0.01 (0.03)	0.02 (0.00)	0.01 (0.01)

**Table 2:** Correlation coefficients between EDA signals from the SP and other locations.

Location	Task	Tonic	Phasic
Chest	Hot	0.526	0.357
	Sandpaper	0.401	0.285
	Pin	0.551	0.244
	Cold	0.069	0.192
	Cotton	0.359	0.234
	Breath	0.041	0.253
Back	Hot	0.462	0.143
	Sandpaper	0.190	0.114
	Pin	0.054	0.084
	Cold	0.105	0.243
	Cotton	0.026	0.068
	Breath	0.219	0.167
Forehead	Hot	0.501	0.110
	Sandpaper	0.105	0.116
	Pin	−0.316	0.211
	Cold	−0.175	−0.057
	Cotton	−0.222	0.087
	Breath	0.080	0.122

## Data Availability

The data are being prepared for publication. At this moment, data are only available from the corresponding author upon request.

## Abbreviations

The following abbreviations are used in this manuscript:

ANS	Autonomic Nervous System
EDA	Electrodermal Activity
SCL	Skin Conductance Level
SCR	Skin Conductance Response
SP	Standard Position
